# Decreased Cezanne expression is associated with the progression and poor prognosis in hepatocellular carcinoma

**DOI:** 10.1186/s12967-015-0396-1

**Published:** 2015-02-01

**Authors:** Jia-hong Wang, Wei Wei, Zhi-xing Guo, Ming Shi, Rong-ping Guo

**Affiliations:** Department of Hepatobilliary Oncology, Sun Yat-sen University Cancer Center; State Key Laboratory of Oncology in South China; Collaborative Innovation Center for Cancer Medicine, 651 Dongfeng Road East, Guangzhou, 510060 P. R. China; Department of Ultrasonics, Sun Yat-sen University Cancer Center; State Key Laboratory of Oncology in South China; Collaborative Innovation Center for Cancer Medicine, Guangzhou, China

**Keywords:** Hepatocellular carcinoma, Cezanne, MMP-9, Aggressiveness, Prognosis

## Abstract

**Background:**

Deubiquitinases, such as CYLD, A20 and Cezanne, have emerged as negative regulators that balance the strength and duration of NF-κB signaling through feedback mechanisms. However, how these serial feedback loops are simultaneously disrupted in cancer remains unclear. The purpose of this study is to investigate the correlation of Cezanne expression with clinicopathological/prognostic value in hepatocellular carcinoma (HCC).

**Methods:**

The expression levels of Cezanne and matrix metallopeptidase 9 (MMP-9) were assessed by immunohistochemistry in 230 HCC specimens. The correlation between expression of Cezanne and MMP-9, clinicopathological/prognostic value in hepatocellular carcinoma was examined.

**Results:**

Cezanne reduction in HCC was significantly associated with larger tumor, satellite nodule, vascular invasion, TNM stage, BCLC stage and early recurrence. Kaplan-Meier analysis showed that Cezanne was a great predictive factor for overall survival (OS) and time to recurrence (TTR). The expression of Cezanne was decreased in TNM and BCLC stage-dependent manner. In addition, Cezanne reduction was associated with poor prognosis in patients subgroups stratified by tumor size, tumor differentiation, TNM stage and BCLC stage. Moreover, Cezanne was negatively associated with MMP-9 among 230 HCC samples. Patients who had Cezanne downregulation, in which cancer cells showed high invasiveness, had shorter TTR and poor OS. Furthermore, the coindex of Cezanne and preoperative serum AFP levels was significantly correlated with OS and TTR.

**Conclusion:**

Cezanne has a pivotal role in tumor progression and prognosis, and may act as a potential prognostic biomarker for survival in HCC patients.

## Introduction

Hepatocellular carcinoma (HCC) is one of the most common solid tumors and prevalent fatal cancers worldwide, especially in East Asia and Sub-Saharan Africa [1,2]. Surgery resection is the preferred curative method, however, a high incidence of postoperative metastasis and recurrence generates a leading challenge as this cancer is most resistant to conventional systemic radiotherapy and chemotherapy [3,4]. In addition, survival may vary widely among HCC patients with the same clinicopathologic features, which is most likely attributable to the heterogeneity of the biological behaviour of tumor cells [5,6]. Therefore, screening the biomarkers for better valuation of recurrence and prognosis of HCC can guide molecular targeting therapy for the improvement of more effective treatments and inhibition of metastasis.

The importance of understanding the molecular biology of HCC has recently gained considerable attention, as molecular targeting therapy has shown encouraging results for many malignancies [7,8]. The key signal transduction pathways implicated in the pathogenesis of liver cancer include the PI3K/Akt/mTOR pathway [9], Wnt/β-catenin signalling cascade [10], and the NF-κB signalling pathway [11,12]. The NF-κB family of transcription factors has an essential role in inflammation and innate immunity. Furthermore, NF-κB is increasingly recognized as a crucial player in many steps of cancer initiation and progression [13]. Studies in cultured cells have demonstrated that NF-κB activity is down-regulated by overexpression of ubiquitin-editing enzyme A20 [14].

Cezanne is a member of the A20 family of deubiquitinating enzymes [15]. Similar to A20, Cezanne has been shown to inhibit NF-κB pathway by deconjugating K63-polyubiquitin chains from RIP-1 and TRAF6 [15,16], which suggests that it may have roles in inhibition of cancer progression. Cezanne is implicated in cancer biology as a 93-kb sequence containing Cezanne was shown to be duplicated in acute lymphoblastic leukemia and Burkitt lymphoma [17]. However, the role of Cezanne in prognosis of HCC patients has not been well clarified.

In this study, we explored the expression of Cezanne in HCC tissues by immunohistochemistry (IHC). Correlation of Cezanne with clinicopathological parameters and prognosis of HCC patients were analysed. Moreover, it has been known that MMP-9 is closely participated in capsular infiltration and metastasis in HCC [18] and serum AFP level is an unfavorable prognostic factor for HCC patients [19]. Therefore, we also assessed the association of Cezanne with MMP-9 protein and investigated the prognostic value of Cezanne combined with serum AFP level in HCC patients.

## Patients and methods

### Patients and specimens

The study was approved by the Institutional Review Board and Human Ethics Committee of Sun Yat-Sen University Cancer Center. Written consent for using the samples for research purposes was obtained from all patients prior to surgery.

All hepatocellular carcinoma samples and adjacent non-tumorous liver tissues were collected from 230 patients who had undergone curative resections from primary HCC between November 2007 and February 2009 at the Department of Hepatobiliary Oncology, Sun Yat-sen University (Guangzhou, China). The eligibility criteria of the current study were as follows: (1) all the samples including HCC and adjacent non-tumorous tissues were histologically confirmed, (2) none of the patients had distant metastasis or received anticancer therapies before the operation, (3) no serious complications or other malignant diseases. The cases were selected consecutively on the basis of availability of resection tissues and follow-up data. The clinicopathological features were obtained from patients’ files (Table 1). Tumor stage was classified according to the 7th Edition tumor-node-metastasis (TNM) classification of the American Joint Committee on Cancer Staging and the Barcelona Clinic Liver Cancer (BCLC) staging system. Overall survival (OS) was defined as from the date of liver resection to the date of death or last follow-up. Time to recurrence (TTR) was measured from the date of surgery until the date of relapse, metastasis, or last follow-up.

**Table 1:** **Patient characteristics**

**Variable**	**No. of patients (%)**
No. of patients	230 (100)
Age: Median [range], y	49 [13–78]
Gender	
Female	26 (11.3)
Male	204 (88.7)
HBsAg	
Negative	15 (6.5)
Positive	215 (93.5)
AFP: Median [range], ng/mL	157.3 [0.6-121000]
GGT: Median [range], U/l	45.0 [3.5-655.5]
Tumor size: Median [range], cm	4.6 [1.0-18.0]
Liver cirrhosis	
No	50 (21.7)
Yes	180 (78.3)
Child-Pugh class	
A	225 (97.8)
B	5 (2.2)
Tumor number	
Single	213 (92.6)
Multiple	17 (7.4)
Satellite nodule	
No	199 (86.5)
Yes	31 (13.5)
Tumor capsule	
No/incomplete	141 (61.3)
Complete	89 (38.7)
Tumor differentiation	
I	14 (6.1)
II	143 (62.2)
III	66 (28.7)
IV	7 (3.0)
Vascular invasion	
No	207 (90.0)
Yes	23 (10.0)
TNM stage	
I	178 (77.4)
II	11 (4.8)
III	41 (17.8)
BCLC stage	
0	20 (8.7)
A	106 (46.1)
B	80 (34.8)
C	24 (10.4)
Recurrence status	
No	105 (45.6)
Early recurrence	85 (37.0)
Late recurrence	40 (17.4)

### IHC staining

A total of 230 HCC tissues and their adjacent non-tumorous samples were used in the IHC analysis. Formalin-fixed, paraffin-embedded specimens from consenting patients were cut in 4 μm sections. After being baked at 60°C for 2 h, the samples were deparaffinized in xylene and rehydrated using a series of graded alcohols. Then, the tissue slides were treated with 3% hydrogen peroxide in methanol for 10 min to exhaust endogenous peroxidase activity. The sections were microwaved antigen retrieval in 0.01 M sodium citrate buffer (pH 6.0) for 30 min, and then preincubated in 10% normal goat serum for 30 min to prevent nonspecific staining. The sections were incubated with the Cezanne mouse monoclonal antibody (working dilution 1:200, Abcam, #ab118387, UK) and MMP-9 rabbit polyclonal antibody (working dilution 1:200, Abcam, #ab38898, UK) overnight at 4°C. The sections were treated with a non-biotin horseradish-peroxidase detection system based on the manufacturer’s instructions (DAKO, Glostrup, Denmark). Assessments of the staining were scores by two experienced pathologists blinded to the patients’ identity and clinical status. In discrepant cases, a pathologist reviewed the cases and reached the consensus.

Both the extent and intensity of immunostaining were taken into consideration when analyzing the data. The intensity of staining was scored from 0 to 3, and the extent of staining was scored from 0% to 100%. The final quantitation of each staining was obtained by multiplying the two scores. Cezanne expression was classified as high expression if the score was higher than the median score of 1.3, if the score was 1.3 or less, the case was classified as low expression. MMP-9 expression was considered high if the score was higher than 1.4.

### Follow-up

The last follow-up was on 30 February 2014. In all the HCC patients (26 females and 204 males), the median follow-up period was 44.5 months, ranging from 3 to 73 months. Recurrence was confirmed by serum α-fetoprotein (AFP) level, abdominal ultrasonography every 2 months, and computed tomography or magnetic resonance imaging or positron emission tomography every 6 months after hepatectomy. The main causes of death were HCC recurrence or complicated cirrhosis of the liver. During the course of follow-up, 125 of 230 HCC patients (54.3%) were found with recurrence and 104 patients (45.2%) died of cancer-related causes. One hundred and twenty-six patients were still alive at the time of the last follow-up report.

### Statistical analysis

The SPSS software package (version 16.0; Chicago, IL, USA) was used for the statistical analysis. The chi-square test was used to analyze the correlation of Cezanne status with clinicopathological features. The Student’s *t*-test was used for comparisons. Pearson χ2 test was applied to analyze the correlation of Cezanne with MMP-9 staining scores. Survival curves were generated using the Kaplan-Meier method, and differences between curves were estimated by the log-rank test. All *P* values were two-sided and *P* values less than 0.05 was considered to be statistically significant.

## Results

### Cezanne expression in HCC

**Figure 1:**
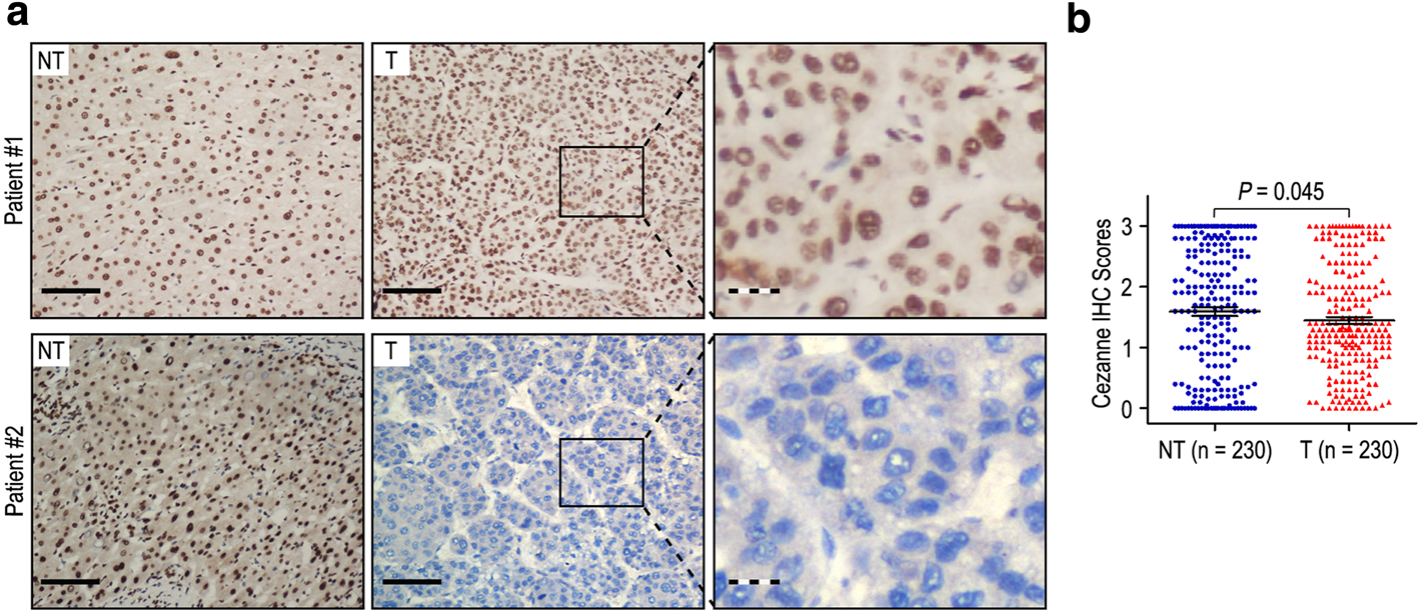
**Cezanne was significantly down-regulated in hepatocellular carcinoma (HCC). (a)** Immunohistochemistry (IHC) assays of Cezanne expression in 230 paired HCC and adjacent non-tumorous tissues. In patient #1, the upper left and middle panel represents high Cezanne expression in adjacent non-tumorous tissue and HCC specimen. The upper right panel represents magnified pictures of boxed area in the corresponding upper middle panel. In patient #2, the lower left panel represents high Cezanne expression in adjacent non-tumorous tissue, while the lower middle panel represents low Cezanne expression in HCC. Lower right panel represents magnified pictures of boxed area in the corresponding lower middle panel. The full line and dotted line scale bar represents 50 μm and 10 μm, respective. **(b)** Cezanne expression levels were compared with HCC and adjacent non-tumorous specimens. Statistical analysis was performed by Paired-Samples *t*-test.

To illuminate the biological significance of Cezanne in HCC, we investigated the immunohistochemical expression of Cezanne in 230 HCC specimens (tumor and matched adjacent non-tumorous tissues). We observed that Cezanne was primarily localized in the nucleus (Figure 1a). We detected low expression of Cezanne in 123/230 (53.5%) of primary HCC specimens, compared with 92/230 (40.0%) in adjacent non-tumorous tissues (*P* = 0.045; Figure 1b). These data indicated that Cezanne expression was significantly lower in HCC tissues than that in adjacent non-tumorous tissues.

### Correlation of Cezanne with clinicopathological variables

To verify the functions of Cezanne in HCC, we correlated Cezanne status in 230 HCC samples with 15 widely recognized clinicopathological features (Table 2). Our results revealed that the low expression of Cezanne in HCC was associated with larger tumor (>5 cm in diameter) (*P* = 0.001), satellite nodule (*P* = 0.001), vascular invasion (*P* = 0.012), TNM stage (*P* = 0.020), BCLC stage (*P* = 0.001) and early recurrence (*P* < 0.001) (Table 2). In contrast, Cezanne expression displayed no correlation with gender, age, AFP, HBsAg, gamma-glutamyltransferase (GGT), liver cirrhosis, tumor number, tumor capsule and tumor differentiation (all *P* > 0.05).

**Table 2:** **Correlation of Cezanne protein expression with clinicopathological parameters**

**Characteristics**	**No. of patients**	**Cezanne expression (%)**		***P*** **-value**
		**Low**	**High**	
Gender				
Female	26	10 (38.5%)	16 (61.5%)	0.103
Male	204	113 (55.4%)	91 (44.6%)	
Age (years)				
≤50	118	62 (52.5%)	56 (47.5%)	0.826
>50	112	61 (54.5%)	52 (45.5%)	
AFP (μg/l)				
≤20	82	44 (53.7%)	38 (46.3%)	0.967
>20	148	79 (53.4%)	69 (46.6%)	
HBsAg				
Negative	15	9 (60.0%)	6 (40.0%)	0.600
Positive	215	114 (53.0%)	101 (47.0%)	
GGT (U/l)				
≤50	122	58 (47.5%)	64 (52.5%)	0.055
>50	108	65 (60.2%)	43 (39.8%)	
Liver cirrhosis				
No	50	26 (52.0%)	24 (48.0%)	0.813
Yes	180	97 (53.9%)	83 (46.1%)	
Tumor size (cm)				
≤5	132	58 (43.9%)	74 (56.1%)	0.001
>5	98	65 (66.3%)	33 (33.7%)	
Tumor number*				
Single	213	113 (53.1%)	100 (46.9%)	0.646
Multiple	17	10 (58.8%)	7 (41.2%)	
Satellite nodule				
No	199	98 (49.2%)	101 (50.8%)	0.001
Yes	31	25 (80.6%)	6 (19.4%)	
Tumor capsule				
No/incomplete	141	77 (54.6%)	64 (45.4%)	0.665
Complete	89	46 (51.7%)	43 (48.3%)	
Tumor differentiation				
I-II	157	88 (56.1%)	69 (43.9%)	0.251
III-IV	73	35 (47.9%)	38 (52.1%)	
Vascular invasion				
No	207	105 (50.7%)	102 (49.3%)	0.012
Yes	23	18 (78.3%)	5 (21.7%)	
TNM stage				
I	178	88 (49.4%)	90 (50.6%)	0.020
II	11	5 (45.5%)	6 (54.5%)	
III	41	30 (73.2%)	11 (26.8%)	
BCLC stage				
0	20	4 (20.0%)	16 (80.0%)	0.001
A	106	52 (49.1%)	54 (50.9%)	
B	80	49 (61.3%)	31 (38.7%)	
C	24	18 (75.0%)	6 (25.0%)	
Early recurrence				
No	145	56 (38.6%)	89 (61.4%)	<0.001
Yes	85	67 (78.8%)	18 (21.2%)	
MMP-9 expression				
Low	118	51 (43.2%)	67 (56.8%)	0.001
High	112	72 (64.3%)	40 (35.7%)	

*Tumor number indicates number of primary tumor mass detected at the time of surgical operation.

### Prognostic value of Cezanne

To further confirm the effect of Cezanne status on OS and TTR in HCC, we performed univariate analysis of traditional clinicopathologic parameters for prognosis. The results revealed that low expression of Cezanne (*P* < 0.001), high AFP level (*P* = 0.016), high GGT level (*P* = 0.001), liver cirrhosis (*P* = 0.006), larger tumor size (*P* < 0.001) and vascular invasion (*P* < 0.001) were unfavourable predictors for OS of HCC patients. In addition, Kaplan-Meier analysis demonstrated that low Cezanne expression (*P* < 0.001), high AFP level (*P* = 0.049), high GGT level (*P* = 0.006), liver cirrhosis (*P* = 0.012), larger tumor size (*P* < 0.001), multiple tumor number (*P* = 0.025), satellite nodule (*P* = 0.009) and vascular invasion (*P* < 0.001) were significantly associated with shorter TTR in HCC patients (Table 3). Moreover, we assessed whether Cezanne could be an independent predictors for OS and TTR in HCC patients. A multivariate Cox model adjusted for AFP, GGT, liver cirrhosis, tumor size, tumor number, satellite nodule, vascular invasion and Cezanne expression was performed. Our data revealed that the Cezanne status was an independent negative prognostic factor for OS (HR = 0.352, 95% CI = 0.226-0.546, *P* < 0.001) and TTR (HR = 0.354, 95% CI = 0.237-0.528, *P* < 0.001) in HCC patients (Table 3).

**Table 3:** **Univariate and multivariate analysis of Cezanne associated with survival and recurrence in HCC patients**

Variables*	**OS**				**TTR**			
	**Univariate**	**Multivariate**			**Univariate**	**Multivariate**		
	***P*** **-value**	***P*** **-value**	**HR**	**95% CI**	***P*** **-value**	***P*** **-value**	**HR**	**95% CI**
Gender (Female vs. Male)	NS	NS			NS	NS		
Age, years (≤50 vs. > 50)	NS	NS			NS	NS		
AFP (ng/mL) (≤20 vs. > 20)	0.016	NS			0.049	NS		
HBsAg (Negative vs. Positive)	NS	NS			NS	NS		
GGT (U/l) (≤50 vs. > 50)	0.001	NS			0.006	NS		
Liver cirrhosis (No vs. Yes)	0.006	0.008	2.216	1.232-3.985	0.012	0.017	1.865	1.115-3.117
Tumor size (cm) (≤5 vs. > 5)	<0.001	0.032	1.616	1.042-2.504	<0.001	0.004	1.866	1.222-2.849
Tumor number (Single vs. Multiple)	NS	NS			0.025	NS		
Satellite nodule (No vs. Yes)	NS	NS			0.009	NS		
Tumor capsule (No/incomplete vs. Complete)	NS	NS			NS	NS		
Tumor differentiation (I-II vs. III-IV)	NS	NS			NS	NS		
Vascular invasion (No vs. Yes)	<0.001	<0.001	2.941	1.706-5.072	<0.001	<0.001	2.471	1.475-4.138
Cezanne (Low *versus* High)	<0.001	<0.001	0.352	0.226-0.546	<0.001	<0.001	0.354	0.237-0.528

*TNM stage and BCLC stage was combined with several clinical indexes such as tumor size, number and tumor thrombus; we did not enter the TNM stage and BCLC stage into multiple analysis with these indexes to avoid any bias in analysis.

GGT gamma-glutamyltransferase, AFP α-fetoprotein, OS overall survival, TTR time to recurrence, NS not significant, HR hazard ratio, CI confidential interval.

**Figure 2:**
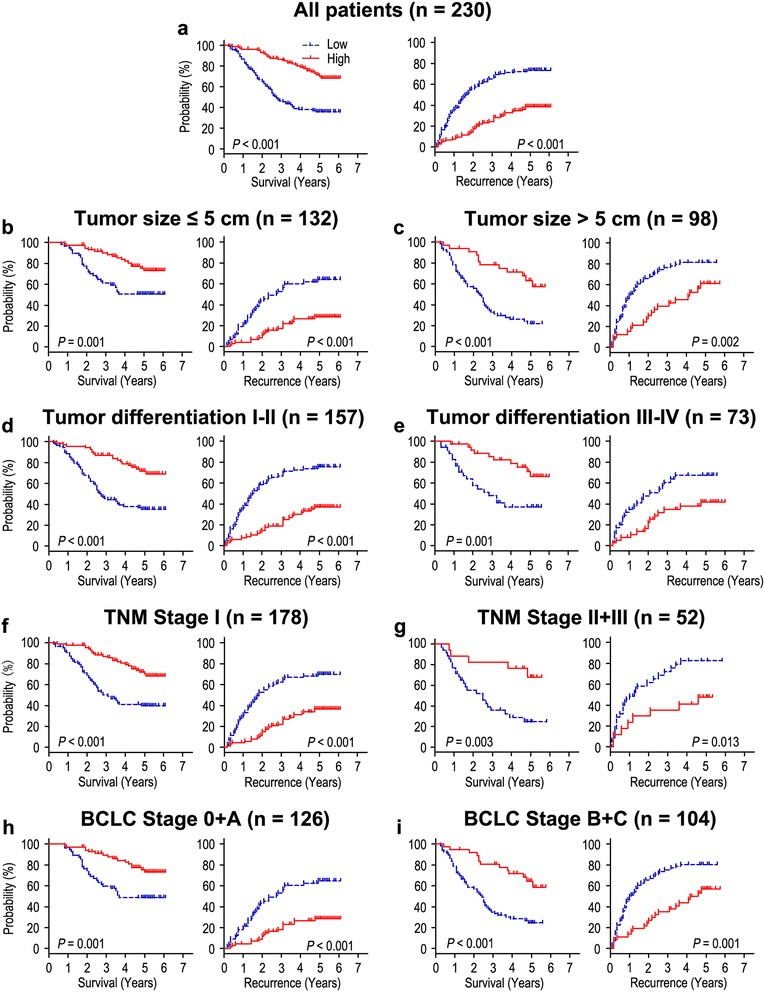
**Overall survival and time to recurrence are shown for patients with HCC.** All patients were stratified according to tumor size, tumor differentiation, TNM classification and BCLC stage. Kaplan-Meier survival estimates and log-rank tests were used to analyze the prognostic significance of Cezanne expression in all patients **(a)** and each subgroup **(b-i)**.

**Figure 3:**
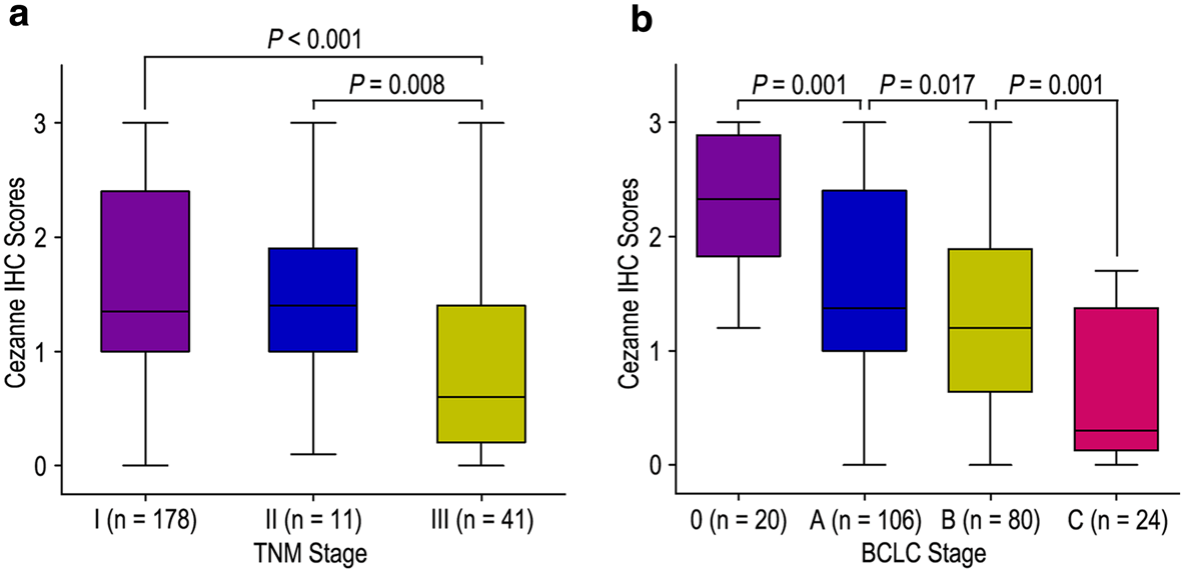
**Comparsion of Cezanne expression by TNM stage and BCLC stage.** Cezanne expression is markedly decreased as tumors progress in TNM stage **(a)** and BCLC stage **(b)**. Statistical analysis was performed by Student’s *t*-test.

Kaplan-Meier analysis revealed that OS and TTR were significantly different in 230 HCC patients based on the expression of Cezanne (both *P* < 0.001) (Figure 2a). The median of OS was 44.5 months, while the median of TTR was 34.0 months. In addition, the median of OS times in Cezanne down-regulation (n = 123) and Cezanne up-regulation (n = 107) HCC patients subgroups were 31.0 months and 57.0 months, while the median of the TTR were 18.0 months and 55.0 months. Furthermore, the 5-year OS and TTR rates of the Cezanne down-regulation group were 37.5% and 26.6%, which were significantly lower than that of the Cezanne up-regulation group (71.6% and 61.4%) (Figure 2a). Moreover, the expression levels of Cezanne in tumors decreased in TNM and BCLC stage-dependent manner, and they were significantly lower in TNM stage III and BCLC stage C tumors than in TNM stage I and BCLC stage 0 tumors (Figure 3).

To further evaluate the prognostic value of Cezanne in different subgroups, patients were stratified according to tumor size (Figure 2b, c), tumor differentiation (Figure 2d, e), TNM stage (Figure 2f, g) and BCLC stage (Figure 2h, i). The Cezanne reduction maintained its prognostic value in predicting shorter OS and TTR in all of these subgroups. Therefore, it suggests that Cezanne may serve as a potential prognostic biomarker for HCC patients in different risk groups.

### Cezanne down-regulation predicts poor prognosis independent of tumor invasiveness

To better understand the clinical significance of Cezanne on progression in HCC, we evaluated the correlation of Cezanne and MMP-9 expression in HCC patients.

**Figure 4:**
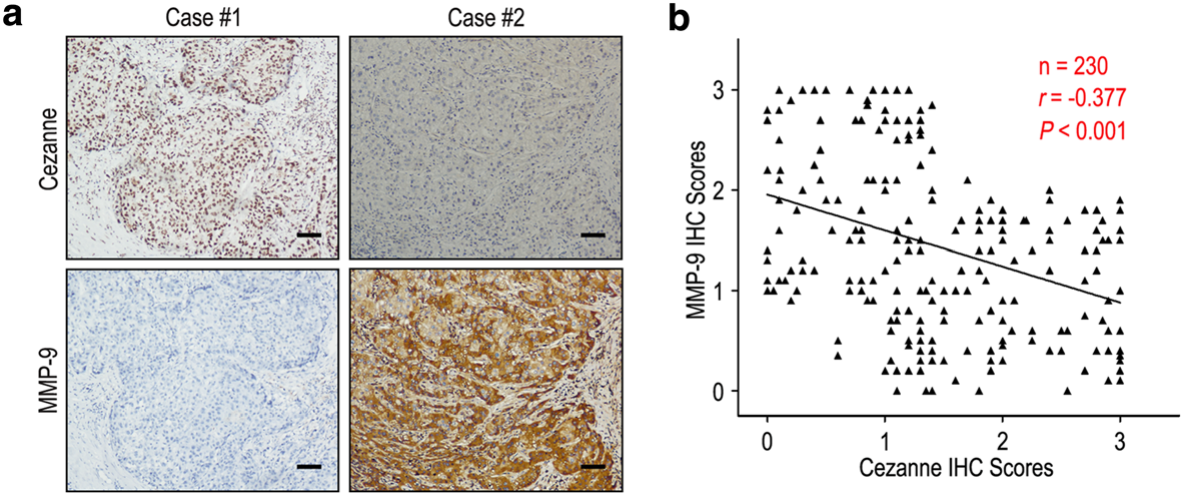
**Cezanne and MMP-9 levels correlated in 230 HCC tissues. (a)** Serial sections of human HCC tissue were subjected to IHC staining with antibodies against Cezanne and MMP-9. In case #1, high expression of Cezanne in HCC tissues was accompanied by the absence of MMP-9. In case #2, low expression of Cezanne was accompanied by elevated MMP-9. The scale bar represents 50 μm. **(b)** Spearman correlation analysis between Cezanne and MMP-9 expression in 230 HCC patients by IHC assays. Cezanne expression was negatively correlated with MMP-9 expression.

The positive rates of Cezanne were only 21.7% and 21.2% in the vascular invasion group and early recurrence group, while there were 49.3% and 61.4% in those without vascular invasion and early recurrence groups (*P* = 0.012 and *P* < 0.001, respectively) (Table 2). In addition, Sixty-seven of 118 (56.8%) patients with low MMP-9 expression had high Cezanne expression, while 72 of 112 patients (64.3%) with high MMP-9 expression had low Cezanne expression (*P* = 0.001, Table 2). Moreover, the relationship of Cezanne and MMP-9 was further confirmed by IHC assays in serial sections of HCC tissues (Figure 4a). The results showed that Cezanne was negatively associated with MMP-9 in 230 HCC samples (*r* = −0.377, *P* < 0.001, Figure 4b).

**Figure 5:**
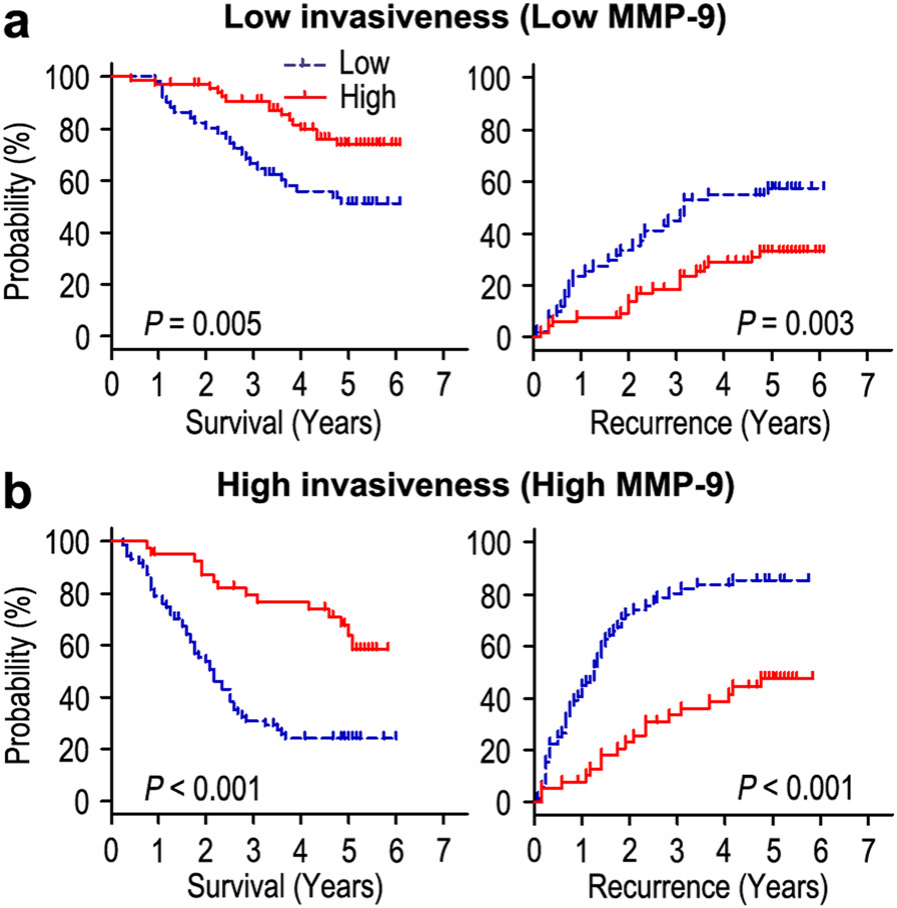
**Overall survival and time to recurrence are shown for patients with low tumor invasiveness (a) and high tumor invasiveness (b).** Kaplan-Meier survival estimates and log-rank tests were used to analyze the association between Cezanne expression and overall survival or time to recurrence in patients with low invasiveness (low MMP-9; n = 118) or high invasiveness (high MMP-9; n = 112).

We further investigated the impact of tumor invasiveness on the prognosis of Cezanne expression in HCC by using MMP-9 marker as an indicator for invasive potential of tumor cells. The HCC patients were classified into either low invasiveness group (low MMP-9 expression; n = 118) or high invasiveness group (high MMP-9 expression; n = 112) based on the MMP-9 expression index. Kaplan-Meier survival curves were then plotted to determine the correlation of Cezanne expression and survival (Figure 5). In the low invasiveness group, Cezanne down-regulation was correlated with poor OS (*P* = 0.005) and shorter TTR (*P* = 0.003) compared with the survival in Cezanne up-regulation patients (Figure 5a). In the high tumor invasiveness group (Figure 5b), patients with Cezanne down-regulation were prone to death (*P* < 0.001) and relapse (*P* < 0.001). Therefore, the expression of Cezanne appears to be a strong postoperative prognostic parameter for patients with HCC independent of tumor invasiveness.

### Prognostic significance of Cezanne combined with serum AFP level on HCC recurrence and survival

**Figure 6:**
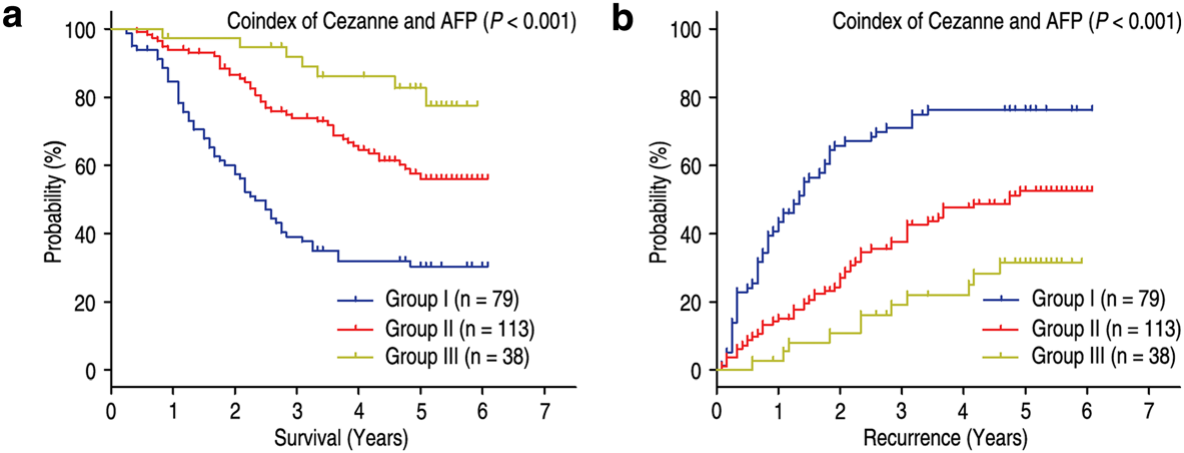
**Kaplan-Meier survival curves according to Cezanne expression combined with serum AFP level in 230 HCC patients. (a)** Overall survival (log-rank *P* < 0.001). **(b)** Time to recurrence (log-rank *P* < 0.001).

It has been known that serum AFP levels are an unfavorable prognostic factor for HCC patients [19]. Univariate analysis indicated that preoperative serum AFP level above 20 ng/mL was significantly associated with shorter OS (*P* = 0.016) and TTR (*P* = 0.049) (Table 3). Therefore, we evaluated the prognostic value of Cezanne expression with serum AFP levels for recurrence and survival of HCC patients. Based on Cezanne expression and serum AFP values, HCC patients were categorized into three groups with different recurrent risks and prognosis: group I, Cezanne (−) and AFP > 20 ng/mL, poor prognosis and high-risk of recurrence; group II with Cezanne (−) and AFP ≤ 20 ng/mL, or Cezanne (+) and AFP > 20 ng/mL, intermediate prognosis and intermediate-risk of recurrence; group III with Cezanne (+) and AFP ≤ 20 ng/mL good prognosis and low-risk of recurrence (Figure 6). Multivariate analysis further demonstrated that the coindex of Cezanne/AFP was an independent prognostic factor for OS (HR = 0.475, 95% CI = 0.348-0.648, *P* < 0.001) and TTR (HR = 0.515, 95% CI = 0.389-0.681, *P* < 0.001) (Table 4).

**Table 4:** **Univariate and multivariate analysis of coindex of Cezanne/AFP associated with survival and recurrence in HCC patients**

**Variables**	**OS**				**TTR**			
	**Univariate**	**Multivariate**			**Univariate**	**Multivariate**		
	***P*** **-value**	***P*** **-value**	**HR**	**95% CI**	***P*** **-value**	***P*** **-value**	**HR**	**95% CI**
Gender (Female vs. Male)	NS	NS			NS	NS		
Age, years (≤50 vs. > 50)	NS	NS			NS	NS		
HBsAg (Negative vs. Positive)	NS	NS			NS	NS		
GGT (U/l) (≤50 vs. > 50)	0.001	NS			0.006	NS		
Liver cirrhosis (No vs. Yes)	0.006	0.010	2.169	1.207-3.897	0.012	0.030	1.756	1.055-2.923
Tumor size (cm) (≤5 vs. > 5)	<0.001	0.037	1.589	1.027-2.458	<0.001	0.007	1.785	1.175-2.710
Tumor number (Single vs. Multiple)	NS	NS			0.025	NS		
Satellite nodule (No vs. Yes)	NS	NS			0.009	NS		
Tumor capsule (No/incomplete vs. Complete)	NS	NS			NS	NS		
Tumor differentiation (I-II vs. III-IV)	NS	NS			NS	NS		
Vascular invasion (No vs. Yes)	<0.001	<0.001	3.027	1.755-5.221	<0.001	<0.001	2.615	1.559-4.386
Coindex of Cezanne/AFP*	<0.001	<0.001	0.475	0.348-0.648	<0.001	<0.001	0.515	0.389-0.681

*Coindex of Cezanne/AFP was combined with Cezanne and AFP, therefore, we did not enter the Cezanne and AFP into univariate and multiple analysis with these indexes to avoid any bias in analysis.

## Disussion

The development of hepatocellular carcinoma (HCC) is closely associated with chronic inflammation caused by viral infection, alcohol consumption, or hepatic metabolic disorders. Evidences have suggested that NF-κB signaling pathway plays an important role in various liver disease and HCC. Persistent NF-κB activation appears to have a central role in the inflammation-fibrosis-cancer axis [20]. NF-κB has been related to initiation, promotion, and progression of HCC [21,22]. Inhibition of NF-κB activity significantly reduced proliferation and invasion of HCC cells as well as downregulated the expression of invasion-related molecules including MMP-9 [23], NF-κB could play an important role in MMP-9 regulation [24,25]. Cezanne expression was shown to be rapidly induced by NF-κB signaling stimulated by TNF-α in a negative feedback manner [16]. In addition, Cezanne negatively modulates NF-κB signaling pathway which has an important role in liver pathology [16,20]. In this study, the expression of Cezanne was explored in 230 HCC tissues by IHC. We found that Cezanne was down-regulated in HCC tissues compared with adjacent non-tumorous tissues. In addition, Cezanne was significantly associated with tumor size, satellite nodule, vascular invasion, TNM stage, BCLC stage and early recurrence. Moreover, the Kaplan-Meier survival analysis showed that the OS and TTR of HCC patients with low Cezanne expression were shorter than those with high Cezanne expression. The prognostic value of Cezanne in different subgroups based on tumor size, tumor differentiation, TNM stage and BCLC stage was also estimated, which appears that Cezanne may serve as a powerful prognostic factor for patients with HCC in different risk groups. According to the results of multivariate analysis, we found that Cezanne down-regulation was an independent predictor for poor OS as well as TTR in HCC. Furthermore, the expression levels of Cezanne were significantly lower in TNM stage III and BCLC stage C tumors than in TNM stage I and BCLC stage 0 tumors. The results indicate that Cezanne has a pivotal role in tumor prognosis, concludes Cezanne could serve as a feasible prognostic biomarker of HCC. Our findings had similar results with previous study. Kanki et al. [26] found that low OTUD7B mRNA expression was correlated with poor prognosis for HCC patients. Moreover, OTUD7B negatively regulated NF-κB signaling pathway, which may be an effective target for antitumor therapy for HCC. Song et al. [27] reported that Cezanne is downregulated in gliomas and correlates inversely with the glioma WHO grading and positively with patients’ survival time. In addition, Cezanne could be used as a tumor suppressor to inhibit the progression of glioma. Our findings and previous observations strongly implicate that Cezanne reduction is involved in the tumor progression and may serve as a prognostic factor for HCC patients.

The degradation of extracellular matrix (ECM) is a signal for the beginning of invasion and metastasis, and matrix metalloproteases are pivotal enzymes that participate in degradation of ECM during invasion and metastasis [28]. MMP-9 was shown to modulate the bioavailability of growth factors and to disrupt cell-cell contacts, dramatically affecting cell proliferation and survival [29]. Arii et al. [18] reported that the expression of MMP-9 mRNA in HCC with capsular infiltration was significantly higher than in HCC without capsular infiltration. In addition, MMP-9 immunoreactivity was the most intense in the HCC cells, particularly in those cells in the marginal areas of the tumorous tissues. MMP-9 is an independent predictor of tumor recurrence and survival in HCC patients [30]. Our results demonstrated that Cezanne was negatively correlated with MMP-9 protein expression. Moreover, the expression of Cezanne was low in patients with vascular invasion and early recurrence (Table 2), the tumor cells with Cezanne overexpression revealed low invasiveness, and low Cezanne expression was a strong predictor of poor prognosis in patients with HCC independent of tumor cell invasiveness (Figure 5). Collectively, Cezanne status in HCC inhibiting tumor progression indicates that Cezanne can be a potential target in cancer therapy.

AFP is a useful tumor-associated antigen for the diagnosis and predicted prognosis of HCC and monitoring metastasis and tumor recurrence in HCC patients with high AFP after hepatectomy [31,32]. However, it is hard to predict the prognosis and metastatic recurrence of normal AFP HCC patients after curative resection. To investigate whether the prognostic value of Cezanne combined with serum AFP level was superior to AFP alone, we divided the HCC patients into three groups according to Cezanne expression and serum AFP level and found that combination of Cezanne and serum AFP level could be used for predicting the risk of tumor recurrence and survival of patients. HCC patients can be classified to different subgroups with different risks of tumor recurrence and prognosis according to Cezanne expression in HCC tissue and preoperative AFP level. Therefore, analysis of Cezanne expression and serum AFP level may help determine whether adjuvant therapy is required after resection.

## Conclusion

In summary, our results revealed that Cezanne may have a pivotal role in tumor invasion and prognosis, and can act as a feasible biomarker for prognostic prediction in HCC. Moreover, the combination of Cezanne with serum AFP level may help to identify the high-risk HCC patients after curative resection and thus aid to select appropriate therapies. It also requires further studies to clarify the underlying biology of Cezanne in the development of HCC.
